# Focus issue on heart failure with preserved left ventricular ejection fraction

**DOI:** 10.1093/ehjci/jeae211

**Published:** 2024-08-16

**Authors:** Otto A Smiseth, Gerald Maurer

**Affiliations:** Institute for Surgical Research, Division of Cardiovascular and Pulmonary Diseases, Oslo University Hospital, Rikshospitalet, University of Oslo, Oslo, Norway; Medical University of Vienna, Spitalgasse 23, 1090 Wien, Austria

**Keywords:** cardiac magnetic resonance, diastolic function, echocardiography, heart failure with preserved ejection fraction

Although heart failure (HF) is a common disorder with high mortality, the diagnosis is often challenging. This is in part because HF symptoms are rather non-specific and may be difficult to differentiate from those caused by obesity, respiratory disease, and other non-cardiovascular conditions. Furthermore, about one-half of all HF patients have normal left ventricular (LV) ejection fraction (EF), and there has been controversy about which alternative measure to use as markers of HF. During the previous two decades, HF phenotyping according to EF has been widely used in clinical studies, and patients were categorized as HF with reduced LV EF ≤ 40%, mildly reduced EF 41–49%, and preserved EF ≥ 50% (HFrEF, HFmrEF, and HFpEF), respectively.^1^ This phenotyping has proven successful in identifying patients who benefit from drug and device therapy, which until a few years ago was limited to those with HFrEF. Following the recent demonstration that sodium–glucose co-transporter 2 (SGLT2) inhibitors are beneficial in HFpEF and with the introduction of medications that target specific cardiomyopathies, HFpEF diagnostics has become increasingly important in clinical practice.^2^

In studies that demonstrated the utility of SGLT2 inhibitors in HFpEF, cardiac imaging was required in the protocols but was limited to measurement of left atrial size or LV mass by echocardiography or cardiac magnetic resonance (CMR).^3,4^ Potentially, more refined phenotyping based on strain imaging and indices of LV diastolic function may be used to optimize patient selection for SGLT2 inhibitor and other HF drugs and should be explored in future studies.

In spite of the role of biomarkers, such as N-terminal pro-B-type natriuretic peptide, imaging continues to play a crucial role for the diagnosis and management of HFpEF. In this focus issue, the most recent imaging approaches for quantification of LV function and diagnosing HFpEF are presented in a series of review articles. One article reviews mechanism behind HFpEF and discusses pathophysiology in a wider context, which includes LV interactions with extra-cardiac organs, including skeletal muscle, adipose tissue, lungs, and kidney.^5^ A separate article reviews how to perform a diagnostic workup for suspected HFpEF, including algorithms for assessing LV filling pressure, and imaging of specific cardiomyopathies.^6^ This focus issue also includes an article that reviews available therapies in HFpEF.^7^

Whereas echocardiography is the dominant clinical imaging technology in HF diagnostics, CMR is the most accurate method for imaging cardiac structure, including myocardial tissue structure, and provides data on myocardial metabolism. A separate article is dedicated to application of CMR in HFpEF diagnostics.^8^ Furthermore, the topics of diastolic function in atrial fibrillation, in arterial hypertension, in non-cardiac pulmonary hypertension and in athletes are reviewed.^9–12^ In addition to the review articles, the focus issue includes several original articles.

We hope this comprehensive collection of articles about imaging in HFpEF will be helpful in the daily clinical work with HF patients and may provide ideas for how to plan and design future studies on management of patients with HFpEF.

## Data Availability

No new data were generated or analysed in support of this research.
